# Reductive cleavage of *N*,*N*′-di-*tert*-butyl­carbodi­imide generates *tert*-butyl­cyanamide ligands, (Me_3_CNCN)^−^, that bind potassium both end-on and side-on in the same single crystal

**DOI:** 10.1107/S205698902000732X

**Published:** 2020-06-09

**Authors:** Amanda B. Chung, Joseph W. Ziller, William J. Evans

**Affiliations:** a Department of Chemistry, University of California, Irvine, CA 92697-2025, USA

**Keywords:** carbodi­imide, cyanamide, urea, 18-crown-6, potassium, crystal structure

## Abstract

*N*,*N*′-Di-*tert*-butyl­carbodi­imide, Me_3_CN=C=NCMe_3_, undergoes reductive cleavage in the presence of the Gd^II^ complex, [K(18-crown-6)_2_][Gd^II^(N*R*_2_)_3_] (*R* = SiMe_3_), to form a new type of ligand, the *tert*-butyl­cyanamide anion, (Me_3_CNCN)^−^. This new ligand can bind metals with one or two donor atoms as demonstrated by the isolation of a single crystal containing potassium salts of both end-on and side-on bound *tert*-butyl­cyanamide anions, (Me_3_CNCN)^−^. The crystal contains [K(18-crown-6)(H_2_O)][NCNCMe_3_-*kN*], in which one (^*t*^BuNCN)^−^ anion is coordinated end-on to potassium ligated by 18-crown-6 and water, as well as [K(18-crown-6)][η^2^-NCNCMe_3_], in which an 18-crown-6 potassium is coordinated side-on to the terminal N—C linkage. This single crystal also contains one equivalent of 1,3-di-*tert*-butyl urea, (C_9_H_20_N_2_O), which is involved in hydrogen bonding that may stabilize the whole assembly, namely, aqua­(*tert*-butyl­cyanamidato)(1,4,7,10,13,16-hexa­oxa­cyclo­octa­deca­ne)potas­sium(I)–(*tert*-butyl­cyanamidato)(1,4,7,10,13,16-hexa­oxa­cyclo­octa­deca­ne)potas­sium(I)–*N*,*N*′-di-*tert*-butyl­carbodi­imide (1/1/1) [K(C_5_H_9_N_2_)(C_12_H_24_O_6_)]·[K(C_5_H_9_N_2_)(C_12_H_24_O_6_)(H_2_O)]·C_9_H_20_N_2_.

## Abstract 2

The crystal structure is reported of a single crystal containing potassium salts of two different *tert*-butyl­cyanamide anions that co-crystallized with one equivalent of 1,3-di-*tert*-butyl urea.

## Chemical context   

**Figure 1:**
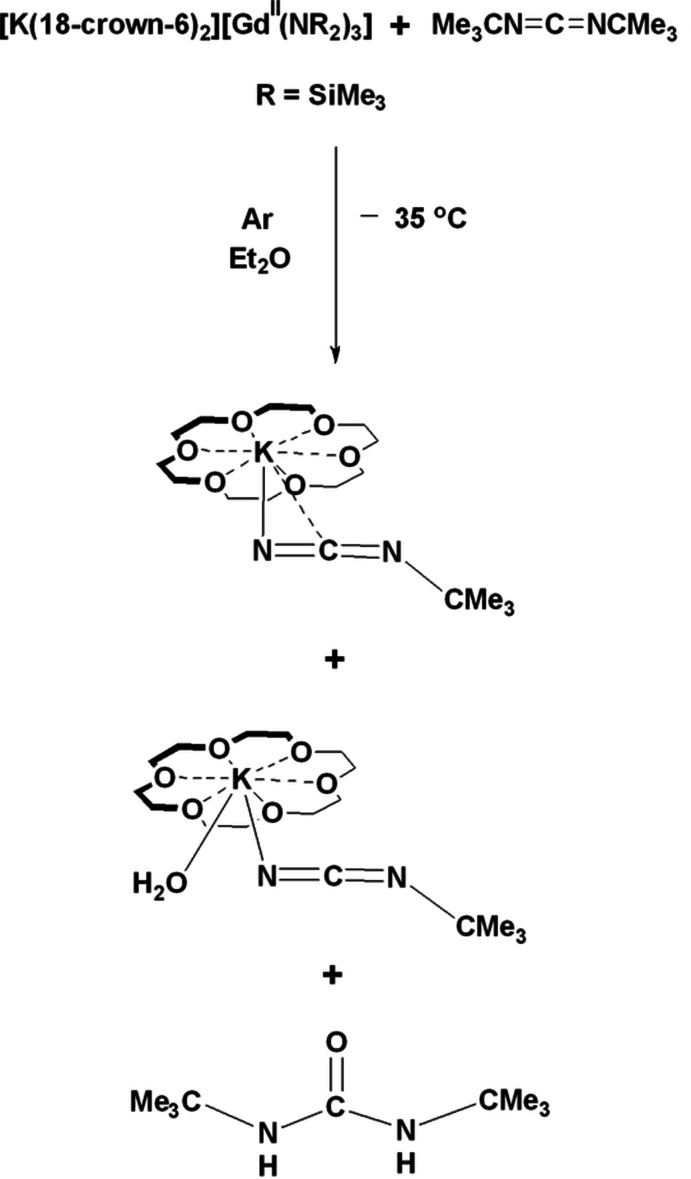
The reaction scheme.

A crystal containing two different potassium 18-crown-6 salts of *tert*-butyl­cyanamide anions, (Me_3_CNCN)^−^, and one equivalent of 1,3-di-*tert*-butyl urea, Fig. 1 ▸, was isolated during the reduction of incompletely dried *N*,*N*′-di-*tert*-butyl­carbodi­imide with [K(18-crown-6)_2_][Gd^II^(N*R*_2_)_3_]. A reductive N—C bond cleavage evidently occurred to remove a *tert*-butyl group from the starting carbodi­imide forming an (Me_3_CNCN)^−^ *tert*-butyl­cyanamide anion that has not previously been observed as a ligand. This reaction could be attributed to the presence of the highly reducing Gd^II^ ion (Ryan *et al.*, 2018 ▸, 2020 ▸). The urea component of the crystal is a formal hydrolysis product of di-*tert*-butyl­carbodi­imide. The presence of water in this reaction system is evident from the fact that one of the 18-crown-6 counter-cations is aqua­ted. The reduction of carbodi­imides with Sm^II^ bis­(tri­methyl­sil­yl)amides, which are not as reducing as Gd^II^, has been known to form oxalamidinates and amidinates (Deacon *et al.*, 2007 ▸).
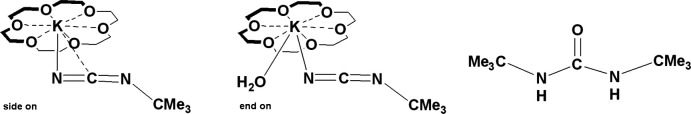


The presence of both end-on and side-on bound *tert*-butyl­cyanamide anions in the same single crystal suggests that these two forms of this ligand are similar in energy. Nature did not pick one over the other during the crystallization process. Hence, this could be a versatile ligand depending on the coordination environment of the cation. In addition, the presence of urea in the single crystal with its hydrogen-bonding connections suggests that this could be a valuable addition to crystallizations to construct complicated assemblies, as found here.

## Structural commentary   

**Figure 2:**
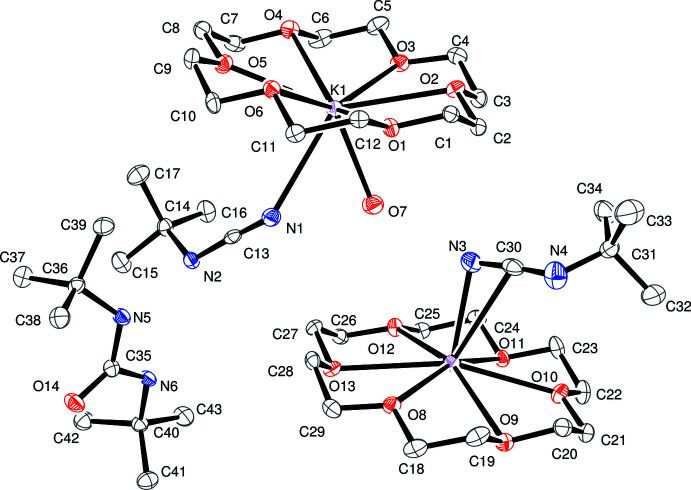
*ORTEP* representation of *tert*-butyl­cyanamide anions bound side-on and end-on and 1,3-di-*tert*-butyl urea, with displacement ellipsoids drawn at the 50% probability level. Hydrogen atoms are omitted for clarity.

An *ORTEP* diagram of the three components of the crystal is shown in Fig. 2 ▸. The two distinct (Me_3_CNCN)^−^ anions have similar metrical parameters as shown in Table 1 ▸. Both anions exhibit N—C—N angles approaching linear, N1—C13—N2 = 176.4 (3)° and N3—C30—N4 = 173.8 (3)°. The (terminal N)—C distances, N3—C30 = 1.185 (4) Å and N1—C13 = 1.179 (3) Å, are in between the 1.13–1.15 Å triple-bond range and the 1.27–1.34 Å double-bond range (Allen *et al.*, 1987 ▸). The Me_3_C—N bonds are also similar, C30—N4 = 1.267 (4) Å and C13—N2 = 1.294 (3) Å, and are in the double-bond range. The C—N—CCMe_3_ angle is 115.3 (2)° for C13—N2—C14 and 120.4 (3)° for C30—N4—C31.

Although the basic structure of the anions is similar, their inter­actions with the potassium counter-cations are different. The K1—N1 distance of 3.027 (2) Å in the component with an end-on bound anion is considerably longer than the 2.699 (2) Å K2—N3 distance of the side-on form. The 3.197 (3) Å K2—C30 distance in the side-on component is considerably longer than either of these K—N distances.

The co-crystallized di-*tert*-butyl urea has metrical parameters identical within experimental error to the three structures in the literature (Gel’bol’dt *et al.*, 2003 ▸, 2005 ▸; So *et al.*, 2014 ▸).

## Supra­molecular features   

**Figure 3:**
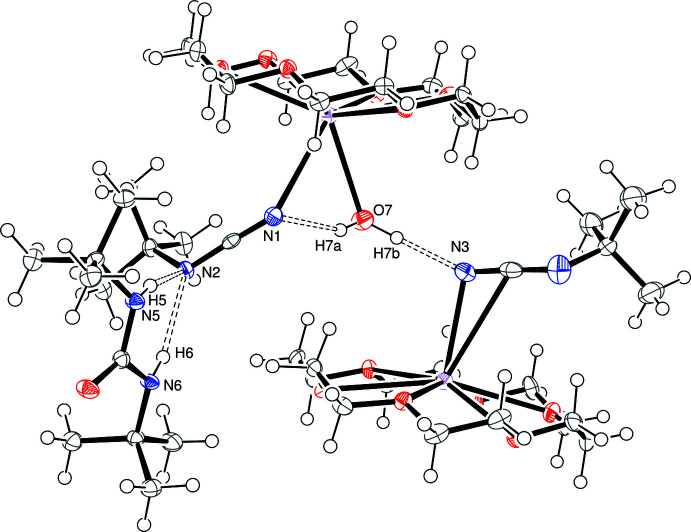
*ORTEP* representation of *tert*-butyl­cyanamide anions bound side-on and end-on and 1,3-di-*tert*-butyl urea, with displacement ellipsoids drawn at the 50% probability level. Hydrogen bonds are shown as dashed lines.

As shown in Fig. 3 ▸, the three components of the crystal are hydrogen bonded (Table 2 ▸). One hydrogen of the water mol­ecule in the [K(18-crown-6)(H_2_O)]^1+^ cation is oriented toward N3, the terminal nitro­gen of the side-on bound cyanamide anion, at distances of 2.26 (3) and 2.29 (3) Å. Both N—H groups on the urea mol­ecule are oriented toward N2, the inter­nal nitro­gen in the end-on bound cyanamide anion, at distances of 2.41 and 2.36 Å.

## Database survey   

A search of the Cambridge Structural Database (CSD, Version 5.40, update of May 2019; Groom *et al.*, 2016 ▸) for (Me_3_CNCN)^−^ anions found no such structures. Three structures of free 1,3-di-*tert*-butyl urea are in the literature. Two structures of the pure compound differ only in the habit of the crystal (Gel’bol’dt *et al.*, 2003 ▸, 2005 ▸) and one structure has the urea co-crystallized with [Ce(L_OEt_)_2_(CO_3_)]·MeC(O)NH_2_ (L_OEt_^−^ = [Co(*η^5^*-C_5_H_5_)-{P(O)(OEt)_2_}_3_] ^−^) (So *et al.*, 2014 ▸).

## Synthesis and crystallization   

*N*,*N*′-Di-*tert*-butyl­carbodi­imide was added dropwise to a dark-blue solution of [K(18-crown-6)_2_][Gd^II^(N*R*_2_)_3_] (*R* = SiMe_3_) (30 mg, 0.026 mmol) in diethyl ether (5 mL) at 238 K. The solution changed from dark blue to colorless after a few minutes. Methyl­cyclo­hexane was layered into the solution and the solution was kept at 238 K, but no crystals were obtained. Solvent was removed to produce a white solid that was dissolved in toluene and placed in a vapor diffusion set up with hexa­nes. After 5 days, small colorless crystals were collected. [K(18-crown-6)_2_][Gd^II^(N*R*_2_)_3_] was synthesized according to a literature procedure (Ryan *et al.*, 2020 ▸).

## Refinement   

Crystal data, data collection and structure refinement details are summarized in Table 3 ▸. H atoms were placed in calculated positions and refined as riding with C—H = 0.98–0.99 and O—H = 0.91 Å and *U*_iso_(H) = 1.2*U*_eq_(C,O) or 1.5*U*_eq_(C-meth­yl).

## Supplementary Material

Crystal structure: contains datablock(s) I. DOI: 10.1107/S205698902000732X/mw2161sup1.cif

Structure factors: contains datablock(s) I. DOI: 10.1107/S205698902000732X/mw2161Isup2.hkl

CCDC reference: 2006987

Additional supporting information:  crystallographic information; 3D view; checkCIF report

## Figures and Tables

**Table 1:** Selected geometric parameters (Å, °)

End-on (Me_3_CNCN)^1−^ component		Side-on (Me_3_CNCN)^1−^ component	
N1—C13	1.179 (3)	N3—C30	1.185 (4)
N2—C13	1.294 (3)	N4—C30	1.267 (4)
N2—C14	1.487 (3)	N4—C31	1.457 (4)
C13—N2—C14	115.3 (2)	C30—N4—C31	120.4 (3)
N1—C13—N2	176.4 (3)	N3—C30—N4	173.8 (3)
C13—N1—K1	132.7 (2)	C30—N3—K2	104.2 (2)
K1—N1	3.027 (2)	K2—N3	2.690 (2)
		K2—C30	3.197 (3)
Urea Component			
O14—C35	1.233 (3)		
N5—C35	1.374 (3)	N5—C36	1.477 (3)
N6—C35	1.367 (3)	N6—C40	1.479 (3)
N6—C35—N5	113.0 (2)		
C35—N5—C36	122.5 (2)	C35—N6—C40	123.3 (2)

**Table 2:** Hydrogen-bond geometry (Å, °)

*D*—H⋯*A*	*D*—H	H⋯*A*	*D*⋯*A*	*D*—H⋯*A*
O7—H7*A*⋯N1	0.81 (4)	2.11 (4)	2.862 (3)	154 (3)
O7—H7*B*⋯N3	0.95 (4)	1.85 (4)	2.788 (4)	173 (4)
N5—H5⋯N2	0.80 (3)	2.26 (3)	3.024 (3)	162 (3)
N6—H6⋯N2	0.82 (3)	2.29 (3)	3.071 (3)	160 (3)

**Table 3:** Experimental details

Crystal data	
Chemical formula	[K(C_5_H_9_N_2_)(C_12_H_24_O_6_)]·[K(C_5_H_9_N_2_)(C_12_H_24_O_6_(H_2_O)]·C_9_H_20_N_2_O
*M*_r_	991.39
Crystal system, space group	Orthorhombic, *P* *n* *a*2_1_
Temperature (K)	88
*a*, *b*, *c* (Å)	21.1326 (10), 8.5470 (4), 29.9188 (14)
*V* (Å^3^)	5403.9 (4)
*Z*	4
Radiation type	Mo *K*α
μ (mm^−1^)	0.24
Crystal size (mm)	0.47 × 0.15 × 0.14

Data collection	
Diffractometer	Bruker SMART APEXII CCD
Absorption correction	Multi-scan (*SADABS*; Bruker, 2014 ▸)
*T*_min_, *T*_max_	0.700, 0.745
No. of measured, independent and observed [*I* > 2σ(*I*)] reflections	42763, 11018, 9556
*R*_int_	0.041
(sin θ/λ)_max_ (Å^−1^)	0.625

Refinement	
*R*[*F* ^2^ > 2σ(*F* ^2^)], *wR*(*F* ^2^), *S*	0.033, 0.065, 1.02
No. of reflections	11018
No. of parameters	614
No. of restraints	1
H-atom treatment	H atoms treated by a mixture of independent and constrained refinement
Δρ_max_, Δρ_min_ (e Å^−3^)	0.19, −0.19
Absolute structure	Flack *x* determined using 3971 quotients [(*I* ^+^)−(*I* ^−^)]/[(*I* ^+^)+(*I* ^−^)] (Parsons *et al.*, 2013 ▸)
Absolute structure parameter	0.015 (13)

Computer programs: *APEX2* (Bruker, 2014 ▸), *SAINT* (Bruker, 2013 ▸), *SHELXL2014/7* (Sheldrick, 2015 ▸), *SHELXTL* (Sheldrick, 2008 ▸), *ORTEP-3 for Windows* (Farrugia, 2012 ▸) and *publCIF* (Westrip, 2010 ▸).

## supplementary crystallographic information

### Crystal data

**Table:**

[K(C_5_H_9_N_2_)(C_12_H_24_O_6_)]·[K(C_5_H_9_N_2_)(C_12_H_24_O_6_(H_2_O)]·C_9_H_20_N_2_O	*D*_x_ = 1.219 Mg m^−^^3^
*M**_r_* = 991.39	Mo *K*α radiation, λ = 0.71073 Å
Orthorhombic, *P**n**a*2_1_	Cell parameters from 9917 reflections
*a* = 21.1326 (10) Å	θ = 2.4–26.2°
*b* = 8.5470 (4) Å	µ = 0.24 mm^−^^1^
*c* = 29.9188 (14) Å	*T* = 88 K
*V* = 5403.9 (4) Å^3^	Prism, colorless
*Z* = 4	0.47 × 0.15 × 0.14 mm
*F*(000) = 2152	

### Data collection

**Table:**

Bruker SMART APEXII CCD diffractometer	9556 reflections with *I* > 2σ(*I*)
Radiation source: fine-focus sealed tube	*R*_int_ = 0.041
φ and ω scans	θ_max_ = 26.4°, θ_min_ = 1.4°
Absorption correction: multi-scan (SADABS; Bruker, 2014)	*h* = −26→26
*T*_min_ = 0.700, *T*_max_ = 0.745	*k* = −10→10
42763 measured reflections	*l* = −37→36
11018 independent reflections	

### Refinement

**Table:**

Refinement on *F*^2^	Secondary atom site location: difference Fourier map
Least-squares matrix: full	Hydrogen site location: mixed
*R*[*F*^2^ > 2σ(*F*^2^)] = 0.033	H atoms treated by a mixture of independent and constrained refinement
*wR*(*F*^2^) = 0.065	*w* = 1/[σ^2^(*F*_o_^2^) + (0.0299*P*)^2^ + 0.3001*P*] where *P* = (*F*_o_^2^ + 2*F*_c_^2^)/3
*S* = 1.02	(Δ/σ)_max_ = 0.001
11018 reflections	Δρ_max_ = 0.19 e Å^−^^3^
614 parameters	Δρ_min_ = −0.19 e Å^−^^3^
1 restraint	Absolute structure: Flack *x* determined using 3971 quotients [(*I*^+^)-(*I*^-^)]/[(*I*^+^)+(*I*^-^)] (Parsons, Flack and Wagner, Acta Cryst. B69 (2013) 249-259).
Primary atom site location: structure-invariant direct methods	Absolute structure parameter: 0.015 (13)

### Special details

**Table:**

Geometry. All esds (except the esd in the dihedral angle between two l.s. planes) are estimated using the full covariance matrix. The cell esds are taken into account individually in the estimation of esds in distances, angles and torsion angles; correlations between esds in cell parameters are only used when they are defined by crystal symmetry. An approximate (isotropic) treatment of cell esds is used for estimating esds involving l.s. planes.
Refinement. A colorless crystal of approximate dimensions 0.140 x 0.150 x 0.474 mm was mounted on a glass fiber and transferred to a Bruker SMART APEX II diffractometer. The APEX2 program package was used to determine the unit-cell parameters and for data collection (45 sec/frame scan time for a sphere of diffraction data). The raw frame data was processed using SAINT and SADABS to yield the reflection data file. Subsequent calculations were carried out using the SHELXTL4 program. The diffraction symmetry was mmm and the systematic absences were consistent with the orthorhombic space groups Pnma and Pna21. It was later determined that space group Pna21 was correct. The structure was solved by direct methods and refined on F2 by full-matrix least-squares techniques. The analytical scattering factors for neutral atoms were used throughout the analysis. Hydrogen atoms involved in hydrogen bonding were located from a difference-Fourier map and refined (x,y,z and Uiso). The remaining hydrogen atoms were included using a riding model.Least-squares analysis yielded wR2 = 0.0649 and Goof = 1.017 for 614 variables refined against 11018 data (0.80 Å), R1 = 0.0326 for those 9556 data with I > 2.0sigma(I). The absolute structure was assigned by refinement of the Flack parameter.

### Fractional atomic coordinates and isotropic or equivalent isotropic displacement parameters (Å^2^)

**Table:**

	*x*	*y*	*z*	*U*_iso_*/*U*_eq_	
K1	0.50347 (2)	0.28541 (6)	0.48666 (2)	0.01627 (12)	
O1	0.43983 (8)	0.5467 (2)	0.44995 (6)	0.0163 (4)	
O2	0.48288 (8)	0.3182 (2)	0.39004 (6)	0.0171 (4)	
O3	0.53709 (8)	0.0453 (2)	0.42504 (6)	0.0189 (4)	
O4	0.50191 (8)	−0.0562 (2)	0.51154 (6)	0.0199 (4)	
O5	0.45852 (9)	0.1706 (2)	0.57014 (6)	0.0209 (4)	
O6	0.40127 (8)	0.4449 (2)	0.53702 (6)	0.0178 (4)	
O7	0.61764 (9)	0.4029 (3)	0.46302 (9)	0.0261 (5)	
H7A	0.6149 (16)	0.453 (4)	0.4860 (13)	0.045 (11)*	
H7B	0.6279 (18)	0.473 (5)	0.4396 (13)	0.061 (12)*	
N1	0.57203 (11)	0.5119 (3)	0.54754 (8)	0.0212 (5)	
N2	0.63884 (10)	0.4839 (2)	0.61486 (7)	0.0165 (5)	
C1	0.42748 (12)	0.5542 (3)	0.40332 (9)	0.0167 (6)	
H1A	0.4227	0.6646	0.3939	0.020*	
H1B	0.3877	0.4980	0.3963	0.020*	
C2	0.48177 (13)	0.4802 (3)	0.37910 (9)	0.0188 (6)	
H2A	0.4766	0.4937	0.3464	0.023*	
H2B	0.5220	0.5302	0.3882	0.023*	
C3	0.53339 (12)	0.2377 (3)	0.36801 (10)	0.0198 (6)	
H3A	0.5746	0.2782	0.3786	0.024*	
H3B	0.5307	0.2550	0.3353	0.024*	
C4	0.52814 (12)	0.0671 (3)	0.37812 (9)	0.0202 (6)	
H4A	0.4859	0.0279	0.3691	0.024*	
H4B	0.5607	0.0082	0.3613	0.024*	
C5	0.53376 (14)	−0.1159 (3)	0.43722 (10)	0.0244 (7)	
H5A	0.5639	−0.1775	0.4190	0.029*	
H5B	0.4906	−0.1564	0.4317	0.029*	
C6	0.54972 (12)	−0.1315 (3)	0.48527 (11)	0.0243 (6)	
H6A	0.5524	−0.2436	0.4934	0.029*	
H6B	0.5914	−0.0827	0.4912	0.029*	
C7	0.51583 (14)	−0.0632 (3)	0.55809 (10)	0.0244 (7)	
H7C	0.5558	−0.0070	0.5644	0.029*	
H7D	0.5210	−0.1735	0.5675	0.029*	
C8	0.46265 (14)	0.0106 (3)	0.58320 (10)	0.0239 (7)	
H8A	0.4225	−0.0440	0.5764	0.029*	
H8B	0.4704	0.0030	0.6158	0.029*	
C9	0.41284 (13)	0.2559 (3)	0.59489 (10)	0.0228 (7)	
H9A	0.4202	0.2417	0.6273	0.027*	
H9B	0.3698	0.2174	0.5878	0.027*	
C10	0.41837 (13)	0.4250 (3)	0.58291 (9)	0.0200 (6)	
H10A	0.3900	0.4881	0.6021	0.024*	
H10B	0.4624	0.4611	0.5877	0.024*	
C11	0.40775 (12)	0.6055 (3)	0.52416 (9)	0.0177 (6)	
H11A	0.4517	0.6410	0.5295	0.021*	
H11B	0.3790	0.6714	0.5423	0.021*	
C12	0.39174 (12)	0.6218 (3)	0.47557 (9)	0.0159 (6)	
H12A	0.3502	0.5727	0.4694	0.019*	
H12B	0.3892	0.7338	0.4674	0.019*	
C13	0.60374 (12)	0.4941 (3)	0.57956 (9)	0.0159 (6)	
C14	0.64677 (12)	0.3233 (3)	0.63316 (9)	0.0174 (6)	
C15	0.69161 (14)	0.3360 (3)	0.67296 (10)	0.0254 (7)	
H15A	0.7337	0.3671	0.6625	0.038*	
H15B	0.6944	0.2345	0.6881	0.038*	
H15C	0.6755	0.4147	0.6939	0.038*	
C16	0.67458 (14)	0.2138 (3)	0.59801 (10)	0.0243 (6)	
H16A	0.6476	0.2145	0.5713	0.036*	
H16B	0.6767	0.1073	0.6101	0.036*	
H16C	0.7172	0.2491	0.5900	0.036*	
C17	0.58367 (13)	0.2559 (3)	0.64942 (11)	0.0265 (7)	
H17A	0.5667	0.3220	0.6733	0.040*	
H17B	0.5904	0.1497	0.6608	0.040*	
H17C	0.5536	0.2528	0.6245	0.040*	
K2	0.76503 (3)	0.71665 (6)	0.41341 (2)	0.01671 (12)	
O8	0.71779 (8)	0.9403 (2)	0.47080 (6)	0.0179 (4)	
O9	0.75617 (8)	1.0449 (2)	0.38524 (6)	0.0187 (4)	
O10	0.80020 (9)	0.8080 (2)	0.32776 (6)	0.0207 (4)	
O11	0.85430 (8)	0.5369 (2)	0.36352 (6)	0.0175 (4)	
O12	0.81650 (8)	0.4450 (2)	0.45097 (6)	0.0170 (4)	
O13	0.77067 (8)	0.6711 (2)	0.51103 (6)	0.0168 (4)	
N3	0.65318 (12)	0.5890 (3)	0.39073 (9)	0.0342 (6)	
N4	0.61560 (13)	0.7606 (3)	0.33083 (9)	0.0340 (6)	
C18	0.71782 (13)	1.1022 (3)	0.45834 (10)	0.0225 (6)	
H18A	0.7595	1.1496	0.4652	0.027*	
H18B	0.6850	1.1593	0.4754	0.027*	
C19	0.70471 (13)	1.1137 (3)	0.40962 (11)	0.0239 (6)	
H19A	0.6648	1.0584	0.4025	0.029*	
H19B	0.6998	1.2249	0.4010	0.029*	
C20	0.74328 (13)	1.0441 (3)	0.33832 (10)	0.0230 (7)	
H20A	0.7378	1.1526	0.3274	0.028*	
H20B	0.7037	0.9857	0.3323	0.028*	
C21	0.79751 (14)	0.9677 (3)	0.31456 (9)	0.0231 (7)	
H21A	0.7914	0.9752	0.2818	0.028*	
H21B	0.8376	1.0211	0.3224	0.028*	
C22	0.84643 (14)	0.7199 (4)	0.30375 (10)	0.0259 (7)	
H22A	0.8894	0.7584	0.3110	0.031*	
H22B	0.8396	0.7314	0.2712	0.031*	
C23	0.84004 (15)	0.5522 (3)	0.31702 (9)	0.0257 (7)	
H23A	0.7964	0.5156	0.3112	0.031*	
H23B	0.8695	0.4870	0.2992	0.031*	
C24	0.84914 (13)	0.3786 (3)	0.37773 (9)	0.0198 (6)	
H24A	0.8784	0.3123	0.3602	0.024*	
H24B	0.8055	0.3405	0.3726	0.024*	
C25	0.86499 (12)	0.3674 (3)	0.42617 (9)	0.0182 (6)	
H25A	0.8676	0.2562	0.4353	0.022*	
H25B	0.9064	0.4173	0.4320	0.022*	
C26	0.82601 (12)	0.4338 (3)	0.49793 (9)	0.0184 (6)	
H26A	0.8653	0.4892	0.5066	0.022*	
H26B	0.8300	0.3228	0.5069	0.022*	
C27	0.77003 (13)	0.5070 (3)	0.52049 (9)	0.0183 (6)	
H27A	0.7304	0.4595	0.5092	0.022*	
H27B	0.7723	0.4895	0.5532	0.022*	
C28	0.71563 (12)	0.7480 (3)	0.52768 (9)	0.0177 (6)	
H28A	0.7119	0.7309	0.5603	0.021*	
H28B	0.6774	0.7050	0.5131	0.021*	
C29	0.72106 (12)	0.9189 (3)	0.51804 (9)	0.0194 (6)	
H29A	0.6862	0.9764	0.5328	0.023*	
H29B	0.7618	0.9595	0.5296	0.023*	
C30	0.63535 (13)	0.6661 (4)	0.36033 (10)	0.0255 (7)	
C31	0.60600 (13)	0.7081 (3)	0.28500 (10)	0.0228 (6)	
C32	0.64918 (17)	0.8036 (4)	0.25414 (12)	0.0444 (9)	
H32A	0.6404	0.9153	0.2580	0.067*	
H32B	0.6413	0.7739	0.2230	0.067*	
H32C	0.6935	0.7825	0.2616	0.067*	
C33	0.53691 (16)	0.7387 (4)	0.27238 (13)	0.0437 (9)	
H33A	0.5093	0.6696	0.2898	0.066*	
H33B	0.5309	0.7183	0.2404	0.066*	
H33C	0.5263	0.8480	0.2789	0.066*	
C34	0.62093 (15)	0.5333 (4)	0.27866 (11)	0.0326 (8)	
H34A	0.6655	0.5141	0.2857	0.049*	
H34B	0.6126	0.5034	0.2476	0.049*	
H34C	0.5941	0.4711	0.2986	0.049*	
O14	0.66354 (8)	0.9028 (2)	0.71848 (6)	0.0233 (4)	
N5	0.59890 (10)	0.7537 (3)	0.67383 (8)	0.0183 (5)	
H5	0.6009 (13)	0.681 (3)	0.6572 (10)	0.017 (8)*	
N6	0.70546 (10)	0.7594 (3)	0.66096 (8)	0.0175 (5)	
H6	0.6967 (14)	0.683 (4)	0.6455 (10)	0.026 (9)*	
C35	0.65670 (12)	0.8105 (3)	0.68721 (9)	0.0172 (6)	
C36	0.54397 (12)	0.7438 (3)	0.70424 (9)	0.0166 (6)	
C37	0.56059 (13)	0.6494 (3)	0.74609 (9)	0.0227 (6)	
H37A	0.5756	0.5453	0.7373	0.034*	
H37B	0.5229	0.6388	0.7649	0.034*	
H37C	0.5939	0.7036	0.7628	0.034*	
C38	0.52015 (13)	0.9054 (3)	0.71749 (10)	0.0221 (6)	
H38A	0.5532	0.9606	0.7341	0.033*	
H38B	0.4824	0.8946	0.7363	0.033*	
H38C	0.5094	0.9649	0.6905	0.033*	
C39	0.49220 (12)	0.6598 (3)	0.67763 (10)	0.0212 (6)	
H39A	0.5072	0.5556	0.6689	0.032*	
H39B	0.4821	0.7204	0.6508	0.032*	
H39C	0.4542	0.6495	0.6962	0.032*	
C40	0.77287 (11)	0.7822 (3)	0.67250 (9)	0.0167 (6)	
C41	0.78957 (13)	0.9557 (3)	0.67113 (10)	0.0222 (6)	
H41A	0.7793	0.9982	0.6416	0.033*	
H41B	0.8349	0.9689	0.6769	0.033*	
H41C	0.7653	1.0115	0.6940	0.033*	
C42	0.78774 (13)	0.7138 (3)	0.71858 (9)	0.0233 (6)	
H42A	0.7624	0.7675	0.7413	0.035*	
H42B	0.8328	0.7281	0.7252	0.035*	
H42C	0.7776	0.6019	0.7188	0.035*	
C43	0.81127 (13)	0.6953 (3)	0.63716 (10)	0.0239 (7)	
H43A	0.7993	0.5845	0.6371	0.036*	
H43B	0.8565	0.7049	0.6440	0.036*	
H43C	0.8027	0.7406	0.6077	0.036*	

### Atomic displacement parameters (Å^2^)

**Table:**

	*U*^11^	*U*^22^	*U*^33^	*U*^12^	*U*^13^	*U*^23^
K1	0.0166 (3)	0.0147 (3)	0.0174 (3)	0.0007 (2)	0.0000 (2)	0.0001 (3)
O1	0.0146 (9)	0.0188 (10)	0.0154 (10)	0.0031 (8)	−0.0010 (7)	−0.0010 (8)
O2	0.0168 (9)	0.0133 (10)	0.0213 (10)	0.0009 (8)	0.0042 (8)	−0.0003 (8)
O3	0.0226 (10)	0.0115 (9)	0.0225 (11)	0.0006 (8)	0.0006 (8)	−0.0018 (8)
O4	0.0190 (10)	0.0175 (10)	0.0232 (11)	0.0026 (8)	−0.0030 (8)	0.0015 (8)
O5	0.0255 (10)	0.0175 (10)	0.0198 (11)	−0.0021 (8)	0.0018 (8)	0.0023 (8)
O6	0.0205 (9)	0.0164 (10)	0.0165 (10)	−0.0008 (8)	−0.0012 (8)	−0.0003 (8)
O7	0.0275 (11)	0.0238 (12)	0.0269 (13)	−0.0030 (9)	0.0043 (10)	−0.0049 (10)
N1	0.0233 (12)	0.0190 (13)	0.0214 (13)	−0.0020 (10)	−0.0021 (10)	0.0034 (10)
N2	0.0216 (12)	0.0129 (12)	0.0151 (12)	0.0006 (9)	−0.0028 (10)	0.0000 (9)
C1	0.0191 (13)	0.0139 (13)	0.0170 (15)	−0.0002 (11)	−0.0045 (11)	0.0023 (11)
C2	0.0230 (14)	0.0182 (15)	0.0151 (15)	−0.0034 (12)	−0.0022 (11)	0.0018 (12)
C3	0.0168 (13)	0.0260 (16)	0.0167 (14)	0.0010 (12)	0.0024 (11)	−0.0025 (12)
C4	0.0151 (13)	0.0216 (15)	0.0239 (16)	0.0008 (11)	0.0016 (12)	−0.0055 (12)
C5	0.0253 (16)	0.0137 (15)	0.0341 (18)	0.0007 (12)	0.0104 (13)	−0.0020 (13)
C6	0.0173 (13)	0.0145 (14)	0.0411 (18)	0.0049 (11)	0.0045 (14)	0.0055 (14)
C7	0.0274 (16)	0.0165 (15)	0.0294 (18)	−0.0044 (12)	−0.0120 (13)	0.0058 (12)
C8	0.0337 (17)	0.0178 (15)	0.0203 (16)	−0.0055 (13)	−0.0080 (13)	0.0063 (12)
C9	0.0250 (15)	0.0291 (17)	0.0145 (15)	−0.0025 (13)	0.0018 (12)	0.0018 (12)
C10	0.0229 (15)	0.0254 (16)	0.0118 (14)	0.0019 (12)	0.0013 (12)	−0.0038 (12)
C11	0.0180 (13)	0.0141 (14)	0.0210 (15)	0.0016 (11)	0.0023 (11)	−0.0018 (11)
C12	0.0147 (12)	0.0122 (13)	0.0209 (15)	0.0028 (10)	0.0007 (11)	−0.0018 (11)
C13	0.0178 (13)	0.0099 (13)	0.0200 (15)	−0.0020 (10)	0.0061 (12)	−0.0008 (11)
C14	0.0201 (14)	0.0163 (14)	0.0157 (14)	0.0023 (11)	−0.0012 (11)	0.0010 (11)
C15	0.0347 (16)	0.0190 (15)	0.0225 (16)	0.0037 (13)	−0.0059 (13)	−0.0006 (12)
C16	0.0318 (16)	0.0193 (15)	0.0218 (16)	0.0067 (13)	−0.0008 (12)	−0.0016 (13)
C17	0.0264 (15)	0.0203 (16)	0.0326 (18)	0.0003 (12)	0.0019 (13)	0.0082 (13)
K2	0.0175 (3)	0.0149 (3)	0.0178 (3)	0.0014 (2)	0.0012 (2)	−0.0003 (3)
O8	0.0219 (9)	0.0121 (9)	0.0197 (10)	0.0001 (8)	0.0014 (8)	−0.0007 (8)
O9	0.0168 (9)	0.0179 (10)	0.0213 (11)	0.0011 (8)	−0.0021 (8)	0.0016 (8)
O10	0.0224 (10)	0.0187 (11)	0.0210 (11)	0.0023 (8)	0.0020 (8)	0.0045 (8)
O11	0.0212 (9)	0.0164 (10)	0.0150 (10)	0.0014 (8)	0.0001 (8)	−0.0018 (8)
O12	0.0172 (9)	0.0179 (10)	0.0158 (10)	0.0054 (8)	−0.0001 (8)	0.0005 (8)
O13	0.0156 (9)	0.0141 (10)	0.0207 (10)	−0.0008 (8)	0.0040 (8)	0.0009 (8)
N3	0.0282 (14)	0.0487 (18)	0.0258 (15)	−0.0134 (13)	−0.0058 (12)	0.0018 (13)
N4	0.0456 (17)	0.0273 (15)	0.0290 (15)	0.0095 (13)	0.0031 (13)	−0.0055 (12)
C18	0.0224 (14)	0.0120 (14)	0.0332 (18)	0.0029 (12)	0.0071 (13)	−0.0024 (12)
C19	0.0182 (13)	0.0171 (14)	0.0364 (18)	0.0029 (11)	0.0034 (13)	0.0056 (14)
C20	0.0251 (15)	0.0194 (15)	0.0246 (17)	−0.0014 (13)	−0.0095 (13)	0.0053 (12)
C21	0.0284 (16)	0.0234 (16)	0.0175 (16)	−0.0044 (13)	−0.0045 (12)	0.0062 (12)
C22	0.0297 (16)	0.0324 (18)	0.0156 (15)	0.0060 (14)	0.0046 (13)	0.0000 (13)
C23	0.0340 (17)	0.0291 (17)	0.0139 (15)	0.0069 (14)	−0.0030 (12)	−0.0050 (13)
C24	0.0210 (14)	0.0140 (14)	0.0246 (16)	0.0024 (11)	0.0016 (12)	−0.0035 (12)
C25	0.0157 (13)	0.0149 (14)	0.0241 (16)	0.0034 (11)	0.0018 (11)	0.0000 (11)
C26	0.0211 (14)	0.0149 (14)	0.0193 (15)	−0.0016 (11)	−0.0053 (11)	0.0016 (11)
C27	0.0223 (14)	0.0146 (14)	0.0180 (15)	−0.0034 (11)	0.0014 (12)	0.0032 (11)
C28	0.0152 (13)	0.0244 (16)	0.0136 (14)	0.0010 (12)	0.0026 (11)	−0.0029 (12)
C29	0.0143 (13)	0.0240 (16)	0.0198 (15)	0.0020 (11)	0.0011 (11)	−0.0069 (12)
C30	0.0178 (14)	0.0332 (17)	0.0255 (17)	−0.0078 (13)	0.0050 (13)	−0.0113 (15)
C31	0.0226 (15)	0.0220 (15)	0.0237 (16)	−0.0038 (12)	0.0001 (12)	0.0036 (13)
C32	0.052 (2)	0.050 (2)	0.031 (2)	−0.0263 (18)	0.0064 (17)	−0.0034 (17)
C33	0.0355 (19)	0.039 (2)	0.056 (2)	−0.0022 (16)	−0.0121 (17)	0.0171 (18)
C34	0.0380 (18)	0.0349 (19)	0.0250 (17)	−0.0015 (15)	0.0008 (14)	−0.0105 (14)
O14	0.0192 (10)	0.0282 (11)	0.0223 (11)	−0.0032 (9)	0.0020 (8)	−0.0114 (9)
N5	0.0161 (12)	0.0217 (13)	0.0172 (13)	−0.0023 (10)	0.0015 (9)	−0.0061 (11)
N6	0.0150 (11)	0.0205 (13)	0.0170 (13)	−0.0029 (10)	0.0009 (9)	−0.0069 (10)
C35	0.0186 (13)	0.0174 (14)	0.0155 (14)	−0.0004 (11)	0.0011 (11)	−0.0011 (11)
C36	0.0148 (13)	0.0182 (15)	0.0169 (14)	−0.0004 (11)	0.0028 (11)	0.0002 (11)
C37	0.0234 (15)	0.0231 (16)	0.0215 (16)	0.0022 (12)	0.0015 (12)	0.0014 (13)
C38	0.0200 (14)	0.0232 (16)	0.0231 (15)	0.0027 (12)	0.0041 (12)	−0.0025 (12)
C39	0.0157 (13)	0.0231 (15)	0.0248 (16)	−0.0011 (11)	0.0011 (12)	−0.0003 (13)
C40	0.0143 (12)	0.0180 (13)	0.0179 (14)	−0.0004 (11)	0.0003 (11)	−0.0009 (12)
C41	0.0172 (14)	0.0221 (16)	0.0273 (17)	−0.0014 (12)	−0.0010 (12)	0.0025 (13)
C42	0.0219 (14)	0.0241 (16)	0.0239 (16)	−0.0014 (13)	−0.0018 (12)	0.0020 (13)
C43	0.0152 (13)	0.0307 (18)	0.0258 (16)	0.0008 (12)	0.0009 (12)	−0.0044 (13)

### Geometric parameters (Å, º)

**Table:**

K1—O7	2.707 (2)	O11—C24	1.422 (3)
K1—O1	2.8291 (18)	O11—C23	1.430 (3)
K1—O5	2.8465 (19)	O12—C26	1.422 (3)
K1—O3	2.8490 (18)	O12—C25	1.429 (3)
K1—O2	2.937 (2)	O13—C28	1.426 (3)
K1—O6	2.9655 (19)	O13—C27	1.431 (3)
K1—O4	3.0134 (19)	N3—C30	1.185 (4)
K1—N1	3.027 (2)	N4—C30	1.267 (4)
K1—H7A	2.75 (4)	N4—C31	1.457 (4)
O1—C1	1.421 (3)	C18—C19	1.487 (4)
O1—C12	1.425 (3)	C18—H18A	0.9900
O2—C2	1.423 (3)	C18—H18B	0.9900
O2—C3	1.430 (3)	C19—H19A	0.9900
O3—C5	1.427 (3)	C19—H19B	0.9900
O3—C4	1.429 (3)	C20—C21	1.498 (4)
O4—C7	1.425 (3)	C20—H20A	0.9900
O4—C6	1.433 (3)	C20—H20B	0.9900
O5—C9	1.419 (3)	C21—H21A	0.9900
O5—C8	1.425 (3)	C21—H21B	0.9900
O6—C10	1.430 (3)	C22—C23	1.494 (4)
O6—C11	1.432 (3)	C22—H22A	0.9900
O7—H7A	0.81 (4)	C22—H22B	0.9900
O7—H7B	0.95 (4)	C23—H23A	0.9900
N1—C13	1.179 (3)	C23—H23B	0.9900
N2—C13	1.294 (3)	C24—C25	1.491 (4)
N2—C14	1.487 (3)	C24—H24A	0.9900
C1—C2	1.497 (4)	C24—H24B	0.9900
C1—H1A	0.9900	C25—H25A	0.9900
C1—H1B	0.9900	C25—H25B	0.9900
C2—H2A	0.9900	C26—C27	1.499 (4)
C2—H2B	0.9900	C26—H26A	0.9900
C3—C4	1.493 (4)	C26—H26B	0.9900
C3—H3A	0.9900	C27—H27A	0.9900
C3—H3B	0.9900	C27—H27B	0.9900
C4—H4A	0.9900	C28—C29	1.493 (4)
C4—H4B	0.9900	C28—H28A	0.9900
C5—C6	1.483 (4)	C28—H28B	0.9900
C5—H5A	0.9900	C29—H29A	0.9900
C5—H5B	0.9900	C29—H29B	0.9900
C6—H6A	0.9900	C31—C33	1.531 (4)
C6—H6B	0.9900	C31—C32	1.533 (4)
C7—C8	1.492 (4)	C31—C34	1.539 (4)
C7—H7C	0.9900	C32—H32A	0.9800
C7—H7D	0.9900	C32—H32B	0.9800
C8—H8A	0.9900	C32—H32C	0.9800
C8—H8B	0.9900	C33—H33A	0.9800
C9—C10	1.494 (4)	C33—H33B	0.9800
C9—H9A	0.9900	C33—H33C	0.9800
C9—H9B	0.9900	C34—H34A	0.9800
C10—H10A	0.9900	C34—H34B	0.9800
C10—H10B	0.9900	C34—H34C	0.9800
C11—C12	1.499 (4)	O14—C35	1.233 (3)
C11—H11A	0.9900	N5—C35	1.374 (3)
C11—H11B	0.9900	N5—C36	1.477 (3)
C12—H12A	0.9900	N5—H5	0.80 (3)
C12—H12B	0.9900	N6—C35	1.367 (3)
C14—C16	1.525 (4)	N6—C40	1.479 (3)
C14—C15	1.526 (4)	N6—H6	0.82 (3)
C14—C17	1.532 (4)	C36—C38	1.522 (4)
C15—H15A	0.9800	C36—C37	1.530 (4)
C15—H15B	0.9800	C36—C39	1.532 (4)
C15—H15C	0.9800	C37—H37A	0.9800
C16—H16A	0.9800	C37—H37B	0.9800
C16—H16B	0.9800	C37—H37C	0.9800
C16—H16C	0.9800	C38—H38A	0.9800
C17—H17A	0.9800	C38—H38B	0.9800
C17—H17B	0.9800	C38—H38C	0.9800
C17—H17C	0.9800	C39—H39A	0.9800
K2—N3	2.690 (2)	C39—H39B	0.9800
K2—O8	2.7565 (18)	C39—H39C	0.9800
K2—O10	2.780 (2)	C40—C41	1.525 (4)
K2—O12	2.7994 (18)	C40—C43	1.526 (4)
K2—O11	2.8541 (18)	C40—C42	1.530 (4)
K2—O9	2.9355 (19)	C41—H41A	0.9800
K2—O13	2.9489 (19)	C41—H41B	0.9800
K2—C30	3.197 (3)	C41—H41C	0.9800
O8—C29	1.427 (3)	C42—H42A	0.9800
O8—C18	1.433 (3)	C42—H42B	0.9800
O9—C20	1.430 (3)	C42—H42C	0.9800
O9—C19	1.435 (3)	C43—H43A	0.9800
O10—C21	1.422 (3)	C43—H43B	0.9800
O10—C22	1.427 (3)	C43—H43C	0.9800
			
O7—K1—O1	91.69 (6)	O12—K2—C30	114.87 (7)
O7—K1—O5	130.88 (7)	O11—K2—C30	103.54 (6)
O1—K1—O5	117.01 (6)	O9—K2—C30	86.08 (7)
O7—K1—O3	82.87 (6)	O13—K2—C30	120.58 (7)
O1—K1—O3	115.85 (6)	C29—O8—C18	112.4 (2)
O5—K1—O3	113.74 (5)	C29—O8—K2	120.69 (15)
O7—K1—O2	80.76 (6)	C18—O8—K2	120.49 (15)
O1—K1—O2	58.13 (5)	C20—O9—C19	110.9 (2)
O5—K1—O2	147.89 (6)	C20—O9—K2	106.81 (15)
O3—K1—O2	57.90 (5)	C19—O9—K2	107.11 (15)
O7—K1—O6	127.68 (6)	C21—O10—C22	113.2 (2)
O1—K1—O6	59.21 (5)	C21—O10—K2	120.98 (16)
O5—K1—O6	57.98 (5)	C22—O10—K2	119.93 (16)
O3—K1—O6	147.32 (5)	C24—O11—C23	111.2 (2)
O2—K1—O6	110.39 (5)	C24—O11—K2	107.76 (14)
O7—K1—O4	115.70 (6)	C23—O11—K2	108.71 (15)
O1—K1—O4	148.83 (5)	C26—O12—C25	112.37 (19)
O5—K1—O4	56.33 (5)	C26—O12—K2	120.47 (15)
O3—K1—O4	57.62 (5)	C25—O12—K2	117.09 (15)
O2—K1—O4	109.51 (5)	C28—O13—C27	112.0 (2)
O6—K1—O4	108.18 (5)	C28—O13—K2	104.60 (14)
O7—K1—N1	59.57 (7)	C27—O13—K2	108.97 (14)
O1—K1—N1	87.49 (6)	C30—N3—K2	104.3 (2)
O5—K1—N1	81.51 (6)	C30—N4—C31	120.3 (3)
O3—K1—N1	136.95 (6)	O8—C18—C19	108.6 (2)
O2—K1—N1	127.03 (6)	O8—C18—H18A	110.0
O6—K1—N1	75.45 (6)	C19—C18—H18A	110.0
O4—K1—N1	118.45 (6)	O8—C18—H18B	110.0
O7—K1—H7A	17.1 (8)	C19—C18—H18B	110.0
O1—K1—H7A	89.6 (7)	H18A—C18—H18B	108.3
O5—K1—H7A	118.1 (8)	O9—C19—C18	109.2 (2)
O3—K1—H7A	99.0 (8)	O9—C19—H19A	109.8
O2—K1—H7A	94.0 (8)	C18—C19—H19A	109.8
O6—K1—H7A	112.8 (8)	O9—C19—H19B	109.8
O4—K1—H7A	120.9 (7)	C18—C19—H19B	109.8
N1—K1—H7A	42.5 (8)	H19A—C19—H19B	108.3
C1—O1—C12	112.14 (19)	O9—C20—C21	108.8 (2)
C1—O1—K1	120.27 (14)	O9—C20—H20A	109.9
C12—O1—K1	119.04 (14)	C21—C20—H20A	109.9
C2—O2—C3	112.0 (2)	O9—C20—H20B	109.9
C2—O2—K1	108.77 (15)	C21—C20—H20B	109.9
C3—O2—K1	107.29 (15)	H20A—C20—H20B	108.3
C5—O3—C4	111.8 (2)	O10—C21—C20	108.5 (2)
C5—O3—K1	121.19 (15)	O10—C21—H21A	110.0
C4—O3—K1	120.57 (14)	C20—C21—H21A	110.0
C7—O4—C6	111.8 (2)	O10—C21—H21B	110.0
C7—O4—K1	106.26 (15)	C20—C21—H21B	110.0
C6—O4—K1	106.97 (14)	H21A—C21—H21B	108.4
C9—O5—C8	113.1 (2)	O10—C22—C23	108.1 (2)
C9—O5—K1	120.52 (15)	O10—C22—H22A	110.1
C8—O5—K1	123.45 (16)	C23—C22—H22A	110.1
C10—O6—C11	110.36 (19)	O10—C22—H22B	110.1
C10—O6—K1	104.42 (14)	C23—C22—H22B	110.1
C11—O6—K1	103.56 (14)	H22A—C22—H22B	108.4
K1—O7—H7A	85 (3)	O11—C23—C22	109.1 (2)
K1—O7—H7B	129 (2)	O11—C23—H23A	109.9
H7A—O7—H7B	108 (4)	C22—C23—H23A	109.9
C13—N1—K1	132.71 (19)	O11—C23—H23B	109.9
C13—N2—C14	115.3 (2)	C22—C23—H23B	109.9
O1—C1—C2	108.4 (2)	H23A—C23—H23B	108.3
O1—C1—H1A	110.0	O11—C24—C25	109.6 (2)
C2—C1—H1A	110.0	O11—C24—H24A	109.8
O1—C1—H1B	110.0	C25—C24—H24A	109.8
C2—C1—H1B	110.0	O11—C24—H24B	109.8
H1A—C1—H1B	108.4	C25—C24—H24B	109.8
O2—C2—C1	108.2 (2)	H24A—C24—H24B	108.2
O2—C2—H2A	110.1	O12—C25—C24	108.3 (2)
C1—C2—H2A	110.1	O12—C25—H25A	110.0
O2—C2—H2B	110.1	C24—C25—H25A	110.0
C1—C2—H2B	110.1	O12—C25—H25B	110.0
H2A—C2—H2B	108.4	C24—C25—H25B	110.0
O2—C3—C4	108.7 (2)	H25A—C25—H25B	108.4
O2—C3—H3A	110.0	O12—C26—C27	107.8 (2)
C4—C3—H3A	110.0	O12—C26—H26A	110.2
O2—C3—H3B	110.0	C27—C26—H26A	110.2
C4—C3—H3B	110.0	O12—C26—H26B	110.2
H3A—C3—H3B	108.3	C27—C26—H26B	110.2
O3—C4—C3	108.5 (2)	H26A—C26—H26B	108.5
O3—C4—H4A	110.0	O13—C27—C26	108.2 (2)
C3—C4—H4A	110.0	O13—C27—H27A	110.1
O3—C4—H4B	110.0	C26—C27—H27A	110.1
C3—C4—H4B	110.0	O13—C27—H27B	110.1
H4A—C4—H4B	108.4	C26—C27—H27B	110.1
O3—C5—C6	108.9 (2)	H27A—C27—H27B	108.4
O3—C5—H5A	109.9	O13—C28—C29	108.7 (2)
C6—C5—H5A	109.9	O13—C28—H28A	109.9
O3—C5—H5B	109.9	C29—C28—H28A	109.9
C6—C5—H5B	109.9	O13—C28—H28B	109.9
H5A—C5—H5B	108.3	C29—C28—H28B	109.9
O4—C6—C5	109.3 (2)	H28A—C28—H28B	108.3
O4—C6—H6A	109.8	O8—C29—C28	108.3 (2)
C5—C6—H6A	109.8	O8—C29—H29A	110.0
O4—C6—H6B	109.8	C28—C29—H29A	110.0
C5—C6—H6B	109.8	O8—C29—H29B	110.0
H6A—C6—H6B	108.3	C28—C29—H29B	110.0
O4—C7—C8	108.6 (2)	H29A—C29—H29B	108.4
O4—C7—H7C	110.0	N3—C30—N4	173.8 (3)
C8—C7—H7C	110.0	N3—C30—K2	54.62 (17)
O4—C7—H7D	110.0	N4—C30—K2	122.8 (2)
C8—C7—H7D	110.0	N4—C31—C33	108.2 (3)
H7C—C7—H7D	108.3	N4—C31—C32	108.7 (2)
O5—C8—C7	108.3 (2)	C33—C31—C32	109.2 (3)
O5—C8—H8A	110.0	N4—C31—C34	112.8 (2)
C7—C8—H8A	110.0	C33—C31—C34	109.3 (2)
O5—C8—H8B	110.0	C32—C31—C34	108.7 (3)
C7—C8—H8B	110.0	C31—C32—H32A	109.5
H8A—C8—H8B	108.4	C31—C32—H32B	109.5
O5—C9—C10	108.6 (2)	H32A—C32—H32B	109.5
O5—C9—H9A	110.0	C31—C32—H32C	109.5
C10—C9—H9A	110.0	H32A—C32—H32C	109.5
O5—C9—H9B	110.0	H32B—C32—H32C	109.5
C10—C9—H9B	110.0	C31—C33—H33A	109.5
H9A—C9—H9B	108.3	C31—C33—H33B	109.5
O6—C10—C9	109.0 (2)	H33A—C33—H33B	109.5
O6—C10—H10A	109.9	C31—C33—H33C	109.5
C9—C10—H10A	109.9	H33A—C33—H33C	109.5
O6—C10—H10B	109.9	H33B—C33—H33C	109.5
C9—C10—H10B	109.9	C31—C34—H34A	109.5
H10A—C10—H10B	108.3	C31—C34—H34B	109.5
O6—C11—C12	109.1 (2)	H34A—C34—H34B	109.5
O6—C11—H11A	109.9	C31—C34—H34C	109.5
C12—C11—H11A	109.9	H34A—C34—H34C	109.5
O6—C11—H11B	109.9	H34B—C34—H34C	109.5
C12—C11—H11B	109.9	C35—N5—C36	122.6 (2)
H11A—C11—H11B	108.3	C35—N5—H5	114 (2)
O1—C12—C11	108.6 (2)	C36—N5—H5	112 (2)
O1—C12—H12A	110.0	C35—N6—C40	123.4 (2)
C11—C12—H12A	110.0	C35—N6—H6	114 (2)
O1—C12—H12B	110.0	C40—N6—H6	117 (2)
C11—C12—H12B	110.0	O14—C35—N6	123.5 (2)
H12A—C12—H12B	108.4	O14—C35—N5	123.5 (2)
N1—C13—N2	176.4 (3)	N6—C35—N5	113.0 (2)
N2—C14—C16	110.9 (2)	N5—C36—C38	111.6 (2)
N2—C14—C15	106.9 (2)	N5—C36—C37	110.7 (2)
C16—C14—C15	110.0 (2)	C38—C36—C37	110.0 (2)
N2—C14—C17	111.5 (2)	N5—C36—C39	105.5 (2)
C16—C14—C17	108.9 (2)	C38—C36—C39	108.9 (2)
C15—C14—C17	108.6 (2)	C37—C36—C39	110.0 (2)
C14—C15—H15A	109.5	C36—C37—H37A	109.5
C14—C15—H15B	109.5	C36—C37—H37B	109.5
H15A—C15—H15B	109.5	H37A—C37—H37B	109.5
C14—C15—H15C	109.5	C36—C37—H37C	109.5
H15A—C15—H15C	109.5	H37A—C37—H37C	109.5
H15B—C15—H15C	109.5	H37B—C37—H37C	109.5
C14—C16—H16A	109.5	C36—C38—H38A	109.5
C14—C16—H16B	109.5	C36—C38—H38B	109.5
H16A—C16—H16B	109.5	H38A—C38—H38B	109.5
C14—C16—H16C	109.5	C36—C38—H38C	109.5
H16A—C16—H16C	109.5	H38A—C38—H38C	109.5
H16B—C16—H16C	109.5	H38B—C38—H38C	109.5
C14—C17—H17A	109.5	C36—C39—H39A	109.5
C14—C17—H17B	109.5	C36—C39—H39B	109.5
H17A—C17—H17B	109.5	H39A—C39—H39B	109.5
C14—C17—H17C	109.5	C36—C39—H39C	109.5
H17A—C17—H17C	109.5	H39A—C39—H39C	109.5
H17B—C17—H17C	109.5	H39B—C39—H39C	109.5
N3—K2—O8	96.89 (7)	N6—C40—C41	110.2 (2)
N3—K2—O10	96.67 (7)	N6—C40—C43	106.6 (2)
O8—K2—O10	118.44 (6)	C41—C40—C43	109.4 (2)
N3—K2—O12	96.11 (7)	N6—C40—C42	111.0 (2)
O8—K2—O12	117.76 (6)	C41—C40—C42	110.4 (2)
O10—K2—O12	119.94 (6)	C43—C40—C42	109.2 (2)
N3—K2—O11	103.34 (7)	C40—C41—H41A	109.5
O8—K2—O11	159.77 (6)	C40—C41—H41B	109.5
O10—K2—O11	59.49 (5)	H41A—C41—H41B	109.5
O12—K2—O11	60.45 (5)	C40—C41—H41C	109.5
N3—K2—O9	105.01 (7)	H41A—C41—H41C	109.5
O8—K2—O9	59.53 (5)	H41B—C41—H41C	109.5
O10—K2—O9	58.94 (5)	C40—C42—H42A	109.5
O12—K2—O9	158.87 (6)	C40—C42—H42B	109.5
O11—K2—O9	113.98 (5)	H42A—C42—H42B	109.5
N3—K2—O13	103.40 (7)	C40—C42—H42C	109.5
O8—K2—O13	59.29 (5)	H42A—C42—H42C	109.5
O10—K2—O13	159.93 (6)	H42B—C42—H42C	109.5
O12—K2—O13	58.48 (5)	C40—C43—H43A	109.5
O11—K2—O13	114.85 (5)	C40—C43—H43B	109.5
O9—K2—O13	114.41 (5)	H43A—C43—H43B	109.5
N3—K2—C30	21.04 (8)	C40—C43—H43C	109.5
O8—K2—C30	95.31 (6)	H43A—C43—H43C	109.5
O10—K2—C30	79.00 (7)	H43B—C43—H43C	109.5
			
C12—O1—C1—C2	177.1 (2)	K2—O9—C20—C21	−62.2 (2)
K1—O1—C1—C2	−35.3 (2)	C22—O10—C21—C20	174.2 (2)
C3—O2—C2—C1	179.7 (2)	K2—O10—C21—C20	−32.9 (3)
K1—O2—C2—C1	−61.9 (2)	O9—C20—C21—O10	65.1 (3)
O1—C1—C2—O2	65.7 (3)	C21—O10—C22—C23	−174.1 (2)
C2—O2—C3—C4	−175.4 (2)	K2—O10—C22—C23	32.7 (3)
K1—O2—C3—C4	65.4 (2)	C24—O11—C23—C22	−179.7 (2)
C5—O3—C4—C3	−179.0 (2)	K2—O11—C23—C22	61.8 (2)
K1—O3—C4—C3	28.9 (2)	O10—C22—C23—O11	−63.6 (3)
O2—C3—C4—O3	−64.1 (3)	C23—O11—C24—C25	179.4 (2)
C4—O3—C5—C6	174.4 (2)	K2—O11—C24—C25	−61.6 (2)
K1—O3—C5—C6	−33.7 (3)	C26—O12—C25—C24	177.2 (2)
C7—O4—C6—C5	−178.2 (2)	K2—O12—C25—C24	−37.1 (2)
K1—O4—C6—C5	−62.2 (2)	O11—C24—C25—O12	67.9 (3)
O3—C5—C6—O4	65.8 (3)	C25—O12—C26—C27	−175.4 (2)
C6—O4—C7—C8	−177.4 (2)	K2—O12—C26—C27	40.2 (2)
K1—O4—C7—C8	66.2 (2)	C28—O13—C27—C26	174.4 (2)
C9—O5—C8—C7	−174.9 (2)	K2—O13—C27—C26	59.1 (2)
K1—O5—C8—C7	24.6 (3)	O12—C26—C27—O13	−66.9 (3)
O4—C7—C8—O5	−62.1 (3)	C27—O13—C28—C29	177.9 (2)
C8—O5—C9—C10	172.6 (2)	K2—O13—C28—C29	−64.2 (2)
K1—O5—C9—C10	−26.2 (3)	C18—O8—C29—C28	175.6 (2)
C11—O6—C10—C9	−178.4 (2)	K2—O8—C29—C28	−32.3 (3)
K1—O6—C10—C9	−67.7 (2)	O13—C28—C29—O8	67.2 (3)
O5—C9—C10—O6	65.5 (3)	C31—N4—C30—K2	119.3 (3)
C10—O6—C11—C12	178.0 (2)	C30—N4—C31—C33	122.2 (3)
K1—O6—C11—C12	66.7 (2)	C30—N4—C31—C32	−119.4 (3)
C1—O1—C12—C11	178.8 (2)	C30—N4—C31—C34	1.2 (4)
K1—O1—C12—C11	30.8 (2)	C40—N6—C35—O14	13.1 (4)
O6—C11—C12—O1	−68.9 (3)	C40—N6—C35—N5	−169.7 (2)
C13—N2—C14—C16	57.6 (3)	C36—N5—C35—O14	−25.0 (4)
C13—N2—C14—C15	177.6 (2)	C36—N5—C35—N6	157.7 (2)
C13—N2—C14—C17	−63.8 (3)	C35—N5—C36—C38	67.4 (3)
C29—O8—C18—C19	−171.7 (2)	C35—N5—C36—C37	−55.5 (3)
K2—O8—C18—C19	36.2 (3)	C35—N5—C36—C39	−174.4 (2)
C20—O9—C19—C18	176.0 (2)	C35—N6—C40—C41	−66.7 (3)
K2—O9—C19—C18	59.8 (2)	C35—N6—C40—C43	174.7 (2)
O8—C18—C19—O9	−65.7 (3)	C35—N6—C40—C42	55.9 (3)
C19—O9—C20—C21	−178.6 (2)		

### Hydrogen-bond geometry (Å, º)

**Table:**

*D*—H···*A*	*D*—H	H···*A*	*D*···*A*	*D*—H···*A*
O7—H7*A*···N1	0.81 (4)	2.11 (4)	2.862 (3)	154 (3)
O7—H7*B*···N3	0.95 (4)	1.85 (4)	2.788 (4)	173 (4)
N5—H5···N2	0.80 (3)	2.26 (3)	3.024 (3)	162 (3)
N6—H6···N2	0.82 (3)	2.29 (3)	3.071 (3)	160 (3)
